# The neural circuits of monogamous behavior

**DOI:** 10.3389/fncir.2022.978344

**Published:** 2022-09-29

**Authors:** María Fernanda López-Gutiérrez, Sara Mejía-Chávez, Sarael Alcauter, Wendy Portillo

**Affiliations:** Instituto de Neurobiología, Universidad Nacional Autónoma de México (UNAM), Querétaro, Mexico

**Keywords:** social monogamy, functional connectivity, multimodal communication, sexual signals, neural plasticity, c-fos

## Abstract

The interest in studying the neural circuits related to mating behavior and mate choice in monogamous species lies in the parallels found between human social structure and sexual behavior and that of other mammals that exhibit social monogamy, potentially expanding our understanding of human neurobiology and its underlying mechanisms. Extensive research has suggested that social monogamy, as opposed to non-monogamy in mammals, is a consequence of the neural encoding of sociosensory information from the sexual partner with an increased reward value. Thus, the reinforced value of the mate outweighs the reward value of mating with any other potential sexual partners. This mechanism reinforces the social relationship of a breeding pair, commonly defined as a pair bond. In addition to accentuated prosocial behaviors toward the partner, other characteristic behaviors may appear, such as territorial and partner guarding, selective aggression toward unfamiliar conspecifics, and biparental care. Concomitantly, social buffering and distress upon partner separation are also observed. The following work intends to overview and compare known neural and functional circuits that are related to mating and sexual behavior in monogamous mammals. We will particularly discuss reports on Cricetid rodents of the *Microtus* and *Peromyscus* genus, and New World primates (NWP), such as the *Callicebinae* subfamily of the titi monkey and the marmoset (*Callithrix spp.*). In addition, we will mention the main factors that modulate the neural circuits related to social monogamy and how that modulation may reflect phenotypic differences, ultimately creating the widely observed diversity in social behavior.

## Introduction

### Neural circuits associated with sexual behavior in monogamous species

Decades of research have led to the hypothesis that pair-bond formation implies an association of the neural representation of the sexual partner with a rewarding valence to generate a selective, long-term preference for socio-sexual interactions with that conspecific (Numan and Young, 2016; Walum and Young, 2018). This proposition states that the expression of these behaviors involves the interaction of sensory and hormonal mechanisms with reward processing brain circuits (Walum and Young, 2018). The study of these interactions can be narrowed into a pair-bonding circuitry, which can be considered as a subnetwork of the social decision-making network (SDMN), a theoretical network made up of thirteen regions that form two interconnected circuits; the social behavioral network and the mesolimbic reward system (Newman, 1999; O’Connell and Hofmann, 2011). The social behavioral network was first described in 1999 in mammals as a hormone-regulated network involved in sexual, aggressive, and parental behaviors that comprise the lateral septum (LS), anterior hypothalamus (AH), ventromedial nucleus of the hypothalamus (VMN), medial amygdala (MeA), bed nucleus of the stria terminalis (BNST), medial preoptic area (mPOA), and the periaqueductal gray (PAG) (Newman, 1999). The second network is the mesolimbic reward system, which is involved in motivation and reward seeking behaviors, the facilitation of reinforcement, decision-making, hedonic processing, and social choice (Wickens et al., 2007; Aragona and Wang, 2009; Báez-Mendoza et al., 2021). This second network consists of the BNST, basolateral amygdala (BLA), caudate putamen (CP), hippocampus (HC), LS, nucleus accumbens (NAcc), ventral pallidum (VP), and the ventral tegmental area (VTA) (O’Connell and Hofmann, 2011). It is proposed that the SDMN regulates adaptive behavior through the interregional coactivity across the network, rather than the activity of the individual region, and is therefore responsible for the processing of relevant social cues and context appropriate behavioral regulation. Thus, the neurobiological mechanisms of social monogamy are necessarily embedded within the SDMN.

Most knowledge about the neural circuitry of social monogamy derives from studies focused on the molecular and genetic mechanisms of pair bonding in the prairie vole (*Microtus ochrogaster*) (Young and Wang, 2004; Ophir, 2017; Walum and Young, 2018). Studies on monogamous non-human primates, with the justification of being closer relatives to humans, have also provided interesting insights into the functional and structural mechanisms underlying monogamous socio-sexual behavior. For instance, a pair-bonding circuit in the titi monkey has already been proposed and may also be involved in other behaviors related to social bonding and attachment (Bales et al., 2017; Savidge and Bales, 2020).

Several reports studying social behavior found that oxytocin (OT) and arginine-vasopressin (AVP) signaling directly impact behaviors associated with pair bonding, such as attachment, social recognition, aggression, parental care, and socio-sexual motivation (Winslow et al., 1993; Williams et al., 1994; Insel and Hulihan, 1995; Insel, 1997; Young et al., 1998; Cho et al., 1999; Pitkow et al., 2001; Bales et al., 2004; Lim and Young, 2004; Lim et al., 2004; Olazábal and Young, 2006; Debiec, 2007; Donaldson et al., 2010; Smith et al., 2010; Keebaugh and Young, 2011; Cavanaugh et al., 2014, 2015, 2018a,2018b; Finkenwirth et al., 2016; Lieberwirth and Wang, 2016; Baxter et al., 2020; Razo et al., 2022). This is why the determination of OT receptor (OTR) and AVP receptor (AVPr) expression patterns in specific brain regions, and the comparison of such circuits between related monogamous and non-monogamous species, have been the standard procedure to elucidate brain regions involved in the modulation of socio-sexual monogamous behavior (Shapiro and Insel, 1992; Insel et al., 1994; Wang et al., 1997b; Hammock and Young, 2004; Smeltzer et al., 2006; Freeman et al., 2014; Freeman and Young, 2016; Smith et al., 2019).

In addition, there is compelling evidence that the interplay and binding of diverse neuroreceptors are key in the modulation of the pair-bonding network. Dopamine (DA) receptors, opioid receptors (κ and μ), estrogen-alpha receptors, and endocannabinoid receptors are known to be substantially involved, while the release of other neurotransmitters like *gamma*-aminobutyric acid (GABA), glutamate, and serotonin has also been reported (Young et al., 2001; Aragona et al., 2006; Smeltzer et al., 2006; Ragen et al., 2015a,b; Cushing, 2016; Larke et al., 2016; Resendez et al., 2016; Putnam et al., 2018; Ulloa et al., 2018; Rothwell et al., 2019). Therefore, accumulating evidence suggests that pair-bonding is a complex social behavior that recruits several brain regions modulated not only by nonapeptide (OT and AVP) and DA signaling (Young et al., 2001; Bales et al., 2017; Ophir, 2017; Walum and Young, 2018) but also by other neuroreceptors and neurotransmitters.

As mentioned previously, pair bonding behavior suggests the interaction and recruitment of brain regions that process sensory-motor, hormonal and reward mechanisms. Some of the brain structures known to be involved in monogamous behavior are the retrosplenial cortex (RSC), HC, and dentate gyrus (DG) of the HC, which are memory-processing regions associated with the encoding of spatial and contextual information (Liu et al., 2014; Lin et al., 2017; Opalka and Wang, 2020). Prefrontal regions like the anterior cingulate cortex (ACC) and the prefrontal cortex (PFC), are related to the processing of relevant external stimuli (salience) (Assadi et al., 2009), and in modulating goal-oriented behavior, respectively (Gill et al., 2010; Ko, 2017). The LS, a subcortical region, is known to be involved in social memory and social reward encoding and integration (Bielsky et al., 2005; Lukas et al., 2013). The VP and NAcc are structures of the ventral striatum associated with reward valence and motivation encoding (Smith et al., 2008; Gárate-Pérez et al., 2019). The VTA is a dopaminergic nucleus also involved in reward and motivation modulation (Steinberg et al., 2014; Faget et al., 2018). The amygdala, which is frequently involved in socio-sexual behavior, can be divided into subregions. The BLA is related to stimulus-outcome representation associations, MeA with social recognition (Ferguson et al., 2001; Ambroggi et al., 2008), and the central amygdala (CeA) with fear and stress response (Davis, 1992; Wilensky et al., 2006). Hypothalamic nuclei like the mPOA are associated with the expression of parental and sexual behavior (Jia et al., 2011), while the BNST has been related to sexual motivation (Marler and Trainor, 2020). The paraventricular nucleus of the hypothalamus (PVN) and the supraoptic nucleus (SON) act as OT and AVP synthesis and release nodes and relevant modulators of social salience in brain circuits (Ross et al., 2009a; Kania et al., 2020; Resendez et al., 2020). The PAG, which has been labeled as an interface between the forebrain and the lower part of the brain stem, has been related to anxiety/defensive behavior and parental care (Kincheski and Carobrez, 2010; Franklin et al., 2017).

These regions, while canonically associated with pair bonding, most likely will not constitute the entirety of the socio-sexual circuit of bonding behavior, and future research will certainly reveal the involvement of additional brain structures due to the complexity implied in this set of behaviors.

### Evolution of the neural circuits of social monogamy

The observed similarities in the functional and structural nature of the brain circuitry in some monogamous mammals has led to the question of the evolutionary origin of social monogamy. It is suggested that, as a mating strategy, social monogamy has evolved independently in most mammalian groups as a consequence of female availability (Lukas and Clutton-Brock, 2013). Specifically, a monogamous mating system, biparental care, and the delayed dispersal of offspring appear to be adapted to the low density of suitable territories and food resources in the prairie and pine vole (*Microtus pinetorum*) environments, whereas plentiful resources in the environments of the montane (*Microtus montanus*) and meadow vole *(Microtus pennsylvanicus*) foster a non-monogamous mating system, uniparental maternal care, and dispersal of the young after they are weaned so they can acquire their territories (Powell and Fried, 1992; Carter et al., 1995). Thus, the similarities of social monogamous behavior between taxa may be a mostly convergent phenomenon and explains why species of the same phylogenetic genus, but with different ecological challenges, have distinct mating strategies. However, at a neurobiological level, it has been shown that the involvement of certain brain regions in the mammal pair-bonding circuit could be somewhat evolutionarily conserved from a functional perspective, though the way they interact may vary depending on the physiological and ecological adaptations for each taxon. Comparisons of nonapeptide expression and its regulation on social behavior between mammalian species have been previously discussed and reviewed in detail, detecting functional analogies (Freeman and Young, 2016; French et al., 2016; Bales et al., 2017). For example, OTR and AVPr distribution studies have revealed that salient sensory cues associated with social interactions may be mostly olfactory in monogamous rodents (Newman and Halpin, 1988). Their sexual behavior-related circuits are oriented toward regions such as the main olfactory bulb (MOB), anterior olfactory nucleus (AON), accessory olfactory bulb (AOB), and the MeA. However, in monogamous New World primates (NWP), socially salient cues may be primarily visual (Freeman et al., 2014; Freeman and Young, 2016), integrating the circuit with visual processing occipital areas like the superior colliculus (SC), pulvinar (Pv), and primary visual cortex, which is consistent with the reliance of primates on visual cues and stimuli (Bales et al., 2017). Moreover, the nucleus basalis of Meynert seems particularly relevant in encoding salience, attention, and novelty, appearing as a central node in the social circuit of primates (Martinez-Rubio et al., 2018; Putnam et al., 2018).

Other sensory systems have already been shown to be relevant for social behavior and mating. For example, the auditory system is disproportionately larger in prairie voles than in other rodents (Campi et al., 2007). Interestingly, OTR expression has been detected in the auditory cortex (Inoue et al., 2022), and complex ultrasonic vocalizations have been observed for mate calling in male voles (Ma et al., 2014) suggesting a relevant role in socio-sexual behavior. Also, common marmosets reportedly rely on scent for socio-sexual communication, and female vocalizations have been observed as a pair-bond maintenance behavior (Evans and Poole, 1983, 1984). Future studies addressing the involvement of other sensory systems in the monogamous circuitry and mate choice would certainly enlarge the scope of the mechanisms involved.

Hence, it may be more useful to compare shared mechanisms between species in more evolutionarily conserved circuit pathways, such as social-reward processing and the interaction of hypothalamic and limbic regions, than comparing them from a sensory-processing level. Similarities in the mechanisms underlying social monogamy have also been discovered at genetic and molecular levels of the organization. Among vertebrate clades, Young and coworkers (Young et al., 2019) evaluated transcriptomes from the forebrain and midbrain of monogamous (*Peromyscus californicus*, *Microtus ochrogaster*) and non-monogamous (*Peromyscus maniculatus*, *Microtus pennsylvanicus*) male Cricetid rodents. They found that mating strategies across vertebrates correlated with neural gene expression patterns, reporting 24 candidate genes associated with monogamy. Monogamous species showed gene ontology term enrichment in processes involved in cell communication, signaling receptor activity, and membrane proteins. Up-regulated genes were involved in neuronal development, cell signaling, synaptic activity, learning and memory, and cognitive functions. In contrast, downregulated genes were associated with RNA polymerase II and AMPA receptor regulation. Their results showed that throughout their evolutionary transition, monogamous mating systems were accompanied by similar changes in gene expression. While the latter study evidenced shared transcriptomic mechanisms across monogamous vertebrates, there were no differences between monogamous and polygamous clades in the AVP or OT expression and in their receptor genes.

Other reports have revealed relevant examples of how social monogamy can diverge from the hypothesis built around a “canonical” nonapeptide-mediated regulation in socio-sexual brain circuitry, which was initially and almost exclusively drawn from vole literature. A study on different species of the Cricetid genus of Peromyscus analyzed AVPr1a brain expression patterns, comparative sequencing, and regulatory regions (Turner et al., 2010). The authors found differentiated levels of AVPr expression on the LS in some species, but these expression levels were not consistent with monogamous and non-monogamous behavioral strategies. No more relationships between the species’ mating system, and any of the analyzed variables associated with this receptor were found. These observations suggest multiple mechanisms for the evolution of monogamy in this rodent genus, in which AVP genetic and microstructural patterns seemed of little contribution to their mating behavior.

Likewise, a report on strepsirrhine primates (lemurs) reached a similar conclusion (Grebe et al., 2021). These mammals share a strong reliance on olfactory perception for social communication with rodents but also have a developed vision with forward-facing eyes and a closer genetic distance to NWP and humans. Similar but diffuse OTR and AVPr expression patterns were detected in olfactory regions when compared to voles, as well as in visual regions when compared to NWP. However, the expected OTR and AVPr-defined pair-bonding circuits similar to what has been observed in other taxa were not found. Monogamous lemur species only had significantly higher OTR binding in the reticular tegmental nucleus and significant stronger AVPr binding in the ventral anterior thalamus, dorsal raphe nucleus, and the PFC in contrast to non-monogamous species. Those results suggest *Eulemur* species developed social monogamy through other neurobiological mechanisms.

It is relevant to mention that both OTR and AVPr expression patterns had initially set a standard in previous research on how a pair-bonding circuit should look, functionally and structurally. However, other neurotransmitter systems may also acquire an alternate (if not complementary) functional role in modulating social salience and pair bond formation, potentially constituting a different pair-bonding neurocircuitry. The neuroendocrine and physiological functions of OT and AVP in mammal reproduction and care of offspring are undoubtedly fundamental. However, it would not be surprising that the plastic properties of the brain, in conjunction with evolutionary pressures, would enable the adaptation of other brain systems (e.g., monoamine or endocannabinoid system) as modulators of social behaviors that could enhance survival through social monogamy. Increasing evidence on the involvement of multiple brain systems in the regulation of social behavior supports this idea (Dölen et al., 2013; Wei et al., 2015, 2017; Larke et al., 2016; Terranova et al., 2016; Xiao et al., 2017, 2018), and contradicts exclusive reliance on OT and AVP for such modulation in monogamous mammalian species. The latter could even turn disadvantageous in generating behavioral diversity and fitness strategies.

Many classical experiments on prairie voles and NWP consisted of pharmacological manipulation of central OTR and AVPr activity, effectively impairing or enhancing pair-bonding behavior (Winslow et al., 1993; Williams et al., 1994; Insel et al., 1995b,^1998^; Insel, 1997; Cho et al., 1999; Donaldson et al., 2010; Smith et al., 2010; Johnson et al., 2016; Simmons et al., 2017; Cavanaugh et al., 2018a,b). However, these reports lacked the technical advantage of knowing the implications of such manipulations over the interaction with other neurotransmitter systems and brain regions comprising the circuitry. Thus, the conclusions on how necessary and sufficient were AVP and OT in prairie vole pair-bonding behavior could not rule out the involvement of other mechanisms and systems. Experiments reporting the inhibition of vole pair-bonding behavior through the pharmacological manipulation of only a single region (NAcc, VP, or LS) (Liu et al., 2001; Liu and Wang, 2003; Lim and Young, 2004; Ross et al., 2009b; Barrett et al., 2013; Anacker et al., 2016) also imply that the whole circuit needs to be intact to function appropriately. However, a recent report has shown widespread expression of OTR throughout the prairie vole brain (Inoue et al., 2022), suggesting extensive but subtle OT neuromodulatory signaling that may require interaction with other neural systems to elicit changes in plasticity associated with bonding behavior. Hence, the complexity of social monogamy has led to the conclusion that no single gene, brain structure, or neurotransmitter system can be the sole modulator of the observed behaviors (Fink et al., 2006). While OT and AVP are undoubtedly involved in the regulation of social bonding and attachment behaviors in mammals, future research must deepen the study of the integration of known neurotransmitter systems in socio-sexual behavior. How socio-sexual behaviors are impacted through simultaneous interaction/inhibition of multiple neurotransmission systems and brain regions in translational studies should provide interesting information on their specific roles in the expression of social monogamy.

Accordingly, it is essential to emphasize that the essence of a pair-bond neural circuit is more functional than structural in the sense that socially salient, partner-related sensory cues must be associated with a rewarding valence to produce a selective preference for the sexual partner and exhibit the appropriate behavior. While initial molecular and genetic studies have been exceptionally useful in setting the necessary grounds to elucidate neural circuits, recent studies have widened our understanding of mating behavior by characterizing the contextual specificity and dynamicity of interactions between brain regions that constitute the functional neural circuits involved in monogamous behavior.

### Brain functional circuits of social monogamy in Cricetid rodents

Studies employing immediate early gene expression have led to important discoveries on the role of brain regions involved in rodent pair bonding. The expression of c-Fos, product of the immediate early gene c-fos, has been widely used because it serves as an indicator of recent neuronal activity and has been used create functional connectivity models during specific social interactions (Gossman et al., 2021). In monogamous voles, c-Fos expression has been assessed in several brain regions of the SMDN and has underpinned important neurobiological mechanisms that underlie pair-bond formation. For example, in both female and male prairie voles, mating has been shown to increase c-Fos expression in the MeA, mPOA, and the BNST, brain regions involved in socio-sexual behavior (Curtis and Wang, 2003; Lim and Young, 2004) and in limbic and reward regions including the VP, NAcc, and the mediodorsal thalamus in male prairie voles (Lim and Young, 2004). c-Fos expression can also be assessed in addition to other experimental methods to unravel the neural circuitry mechanisms that underlie social attachment. In the latter study (Lim and Young, 2004), it was shown that induced over-expression of AVPr1a in the VP was accompanied by an increase in mating-induced c-Fos expression. Also, Northcutt and Lonstein assessed c-Fos expression in tyrosine hydroxylase (TH)-positive cells in the BNST and posterodorsal MeA (pdMeA) of male prairie voles and reported that the c-Fos expression in TH-positive cells decreased after social isolation, whereas mating elicited greater c-Fos expression in both the BNST and the pdMeA (Northcutt and Lonstein, 2009). These findings suggest that dopaminergic activity of the BNST and pdMeA may be a unique neural mechanism through which animals process socially relevant cues and then transmit this information to dopamine-sensitive brain regions necessary for social bonding (Northcutt and Lonstein, 2009). A relationship between OTR signaling and c-Fos expression in prairie vole socio-sexual behavior have also been studied. A study by Johnson and coworkers suggested that OTRs in the NAcc could modulate the activity of some regions that are part of the SDMN in male prairie voles. However, the blockade of OTRs in the NAcc was reported to have no robust effect on sociosexual behaviors and did not modify the activity of the AON, PFC, MeA, or the PVN (Johnson et al., 2017). These findings show that OTR activity in the NAcc during sociosexual interactions does not modulate the patterns of c-Fos activity across the nodes of the SDMN. Instead, these data support the hypothesis that SDMN regulates adaptive behavior through interregional coactivation across its nodes (Newman, 1999; O’Connell and Hofmann, 2011).

A novel application of studying c-Fos expression in brain tissue is the analysis of the simultaneous co-expression in different brain regions, which allows the generation of functional connectivity models. Functional connectivity may be defined as the correlation of activity between anatomically separated brain regions (Friston et al., 1993) and has been mainly described in neuroimaging (Park and Friston, 2013) and electrophysiological recordings (Amadei et al., 2017). Its conceptual application has been extended to the co-expression patterns of immediate early genes (Tanimizu et al., 2017). This connectivity has already been studied between regions of the SMDN during different social interactions in pair-bonded male prairie voles through c-Fos expression patterns (Gossman et al., 2021). The social interactions assessed in this study were the exposure to their pair bonded partner, a same-sex stranger, and an opposite-sex stranger. Consistent with the literature, the study showed that pair-bonded male prairie voles displayed selective affiliation toward their partner and selective aggression toward both opposite-sex and same-sex strangers (Wang et al., 1997a; Gobrogge et al., 2007). Additionally, different social encounters induced context-dependent c-Fos activity in some regions of the SDMN. Specifically, evoked brain activity upon exposure to their pair bonded partner was reported in the mPOA, BNST, LS, and VP, consistent with the findings of Gobrogge and coworkers (Gobrogge et al., 2007). Moreover, the mPOA, BNST, LS, and the VP were also activated upon exposure to a same-sex stranger, but only the VMH activity increased upon exposure to all social encounters (Gossman et al., 2021). In addition to the analysis of c-Fos brain induced activity, Gossman et al. (2021) also analyzed coactivation patterns across regions using correlational models, hierarchical cluster analysis, and graph theory-derived network modeling. The partner-related network incorporated the AH, LS, mPOA, and the VP regions suggested to compose the social behavioral network (Newman, 1999; O’Connell and Hofmann, 2011) and involved in affiliative behaviors in the prairie vole (Curtis and Wang, 2003; Cushing et al., 2003; Lim and Young, 2004; Gossman et al., 2021). However, the activity of the AH has also been associated with aggression toward unfamiliar female or male strangers in male prairie voles (Wang et al., 1997a; Gobrogge et al., 2007). Another set of regions involved in the partner-related network were the NAcc, MeA, BLA, and mPAO, which have also been incorporated into an alternative network described as the pair bonding network (Johnson et al., 2016). These studies show that identifying interconnected circuits can provide insight into how brain regions work as a functional network during specific social interactions, including affiliative behaviors.

A relevant example of how functional connectivity allows the study of behavioral correlates with neural activity on socio-sexual circuits can be shown in a study involving the mPFC-NAcc pathway in female prairie voles (Amadei et al., 2017). The circuit dynamics of these two regions were studied through free-moving electrophysiological recordings during sexual interactions (Amadei et al., 2017). The increase of functional modulation from the mPFC to the NAcc as a consequence of mating correlated with the predisposition and display of huddling, a characteristic affiliative behavior in this rodent (Salo et al., 1993). During a restricted cohabitation protocol that prevented mating, this pathway was optogenetically activated when female voles interacted with a caged male. The following day, these females displayed a greater preference for the partner than for the stranger, demonstrating that the activation of a circuit related to an affiliative behavior can bias into the formation of a pair bond. While mating is undoubtedly a relevant enhancer in pair-bond formation in the prairie vole, the manifestation of huddling was equally important, since both behaviors modulated a reward codification and response that was necessary for pair-bond maintenance.

With respect to the analysis of functional neural circuits at a larger scale in the prairie vole, functional magnetic resonance imaging (fMRI) has been the method of choice and has successfully detected the brain’s functional response to sensory stimulation (Yee et al., 2016) and resting-state networks that are analogous to other rodents and humans (Ortiz et al., 2018). Though fMRI alone cannot distinguish the activity of specific ligands in the brain, it is an exceptionally powerful technique for characterizing brain networks and allows studying functional connectivity between brain regions. Longitudinal fMRI resting-state networks of prairie voles were analyzed before and after pair bonding, and functional interactions were assessed in 16 different regions relevant for vole socio-sexual behavior at 24 h and 2 weeks after the onset of cohabitation (López-Gutiérrez et al., 2021). Several functional brain networks were found, in which a cortico-striatal functional network integrating the VP, MeA, LS, VTA, RSC, BLA, PVN, NAcc, ACC, and the DG correlated higher functional connectivity with how quick a male or female vole displayed huddling during cohabitation (huddling latency), also predicting the onset of affiliative behavior as partially shown by Amadei and colleagues (Amadei et al., 2017). Following 24 h of cohabitation, a network including the LS, NAcc, medial prefrontal cortex (mPFC), MeA, VP, MOB, and DG correlated with the amount of social interaction. The longer a pair-bonded vole would interact with a conspecific of the opposite sex was related to lower functional connectivity, suggesting a neural correlate of after-bonding approach-avoidance behavior. Additionally, region interactions involving connectivity between the LS, VP, BLA, NAcc, and RSC correlated with partner preference in both male and female voles, reflecting how the same regions can participate in pair-bond formation and maintenance by interacting in a temporally dynamic manner (López-Gutiérrez et al., 2021).

Also, the LS appeared as a social behavior modulation hub, integrating information from cortical, limbic, and social memory structures that may be involved with the formation of new social and contextual memories related to the partner, as predicted by Alexander G. Ophir (2017). The importance of the LS as a node in socio-sexual functional networks is also evident in NWP. This evidence integrates previous findings reported with other methods and further confirms that social circuits in the prairie vole vary individually and the relationship between regions of the SDMN is temporally and behavior-wise context-dependent, so studies that further address the specificity of such interactions are of utter importance.

In López-Gutiérrez et al. (2021), male and female voles from the same population displayed individual variability in prosocial behavior and partner preference, so differences in the functional connectivity of socio-sexual behavior circuits correlate with the alternate mating tactics observed in individuals of this rodent species, in which nonapeptide (AVP and OT) receptor density variation in the NAcc, RSC, and VP has been found relevant in the diversity of such tactics (Lim et al., 2004; Ophir et al., 2008b; King et al., 2016).

Concerning prairie vole mating tactics, individuals that display promiscuous behavior are often labeled as “wanderers,” which generally exhibit better spatial and navigational memory and overlap several home ranges, increasing the probability of opportunistic mating. In opposition, voles labeled as “residents,” have a more monogamous strategy, often displaying territorial behavior and mate guarding for mating success (Getz et al., 2005; Ophir et al., 2008b; Phelps et al., 2017; Madrid et al., 2020; Forero and Ophir, 2022). Nonetheless, some individuals may not necessarily fit into these categories and, as later explained in more detail, socio-sexual circuits are subjected to modulation by several genetic and external factors that enable behavioral adaptation and, more broadly, adaptation to ecological pressures in the environment. At a population level, this has already been shown. Through fMRI and diffusion-weighted magnetic resonance imaging (DWI), differences in the microarchitecture (Ortiz et al., 2020) and functional network topology (Ortiz et al., 2021) of socio-sexual circuits have been found between male prairie voles from two populations that had distinct levels of prosocial behavior. Male voles with greater prosocial behavior had a higher fraction of anisotropy (FA), a measure of water diffusion that provides information of brain tissue density and organization in regions such as the PVN, BNST, dorsal raphe nucleus and anterior thalamic nucleus. The PVN, BNST, and MeA also exhibited more functional connections in prosocial males, suggesting that both brain architecture and functional connectivity may be associated with behavioral differences (Ortiz et al., 2020). Further, male voles from a prosocial population were shown to have a higher global brain functional connectivity in regions related to socio-sexual behavior. The BNST and AH appeared as hubs (i.e., regions with high levels of connections). A higher connectivity between olfactory regions and social behavior regions was also detected in prosocial male voles, with exclusive connections involving the AH, mPOA, PVN and the NAcc (classified as a hub), in comparison to male voles of another population with lower prosocial behavior (Ortiz et al., 2021). These studies postulated that in order to fully understand how neural circuits influence socio-sexual behavior, they must consider the ecological context of the species, since brain plasticity is open to influences from the molecular and genetic levels to the environmental and populations levels (Madrid et al., 2020). While this has been more studied in rodents, the prairie vole in particular, current research on the NWP should focus on how individual neural and behavioral correlates contribute in a socio-ecological context.

### Brain functional circuits of social monogamy in new world primates

In monogamous NWP, imaging studies have made considerable advances in identifying the functional interactions of the socio-sexual behavior brain circuitry. In male titi monkeys, positron emission tomography (PET) scans co-registered with structural magnetic resonance imaging were employed to indirectly measure changes in neural plasticity through the comparison of regional and global levels of cerebral glucose metabolism. Through the administration of [^18^F]-fluorodeoxyglucose (FDG) before and 48 h after pair bonding, significant changes were observed in the NAcc, VP, mPOA, MeA, SON, and LS (Bales et al., 2007). A follow-up study showed long-term increased whole-brain FDG uptake within 1 week of pairing, and increased uptake in the NAcc and VP (Maninger et al., 2017a). When treated with intranasal OT during the juvenile period, increased engagement and behavior was induced; a month later, changes in FDG uptake were detected in the SDMN (NAcc, LS, ACC, CeA, HC, PVN, and SON) (Arias Del Razo et al., 2020). In another study, a neural correlate of “jealousy” was described in male titi monkeys. Previously paired males that were placed alone in a cage had visual access to another cage with their female pair mate next to a male stranger. Compared to the control condition, they showed increased FDG uptake in the right LS, left posterior cingulate cortex (PCC), left ACC, and decreased uptake in the MeA, putatively detecting a circuit related to mate guarding (Maninger et al., 2017b). DA receptor binding was also studied through PET scans before and after pair bonding and significant differences were found in the LS of male titi monkeys, demonstrating that neuroplasticity of the DA system facilitated mate guarding behavior (Hostetler et al., 2017). Accumulating evidence shows that the LS may be a relevant network hub in pair-bonding formation, pair-bond maintenance, and in the expression of behaviors like mate guarding and separation/reunion in the NWP. The co-localization of key neurotransmitters for reward and social behavior, particularly DA, OT, and opioid receptors (Freeman et al., 2014; Ragen et al., 2015a; Putnam et al., 2018), further support this role. Functional network changes in the marmoset monkey have yet to be studied and would be valuable as a comparative study to comprehend the level of functional conservation of neural correlates and behavior interactions in NWP models.

### Differences and similarities in circuits of socially monogamous mammals

We have summarized the similarities and differences of known circuit-level interactions of particular behaviors in some of the monogamous species covered in this review (Table 1). Unfortunately, there is not enough evidence available for all species. However, data suggests that the NAcc is a central node in most behaviors, likely modulating cue-evoked behavioral responses associated with a reward valence (Stern and Passingham, 1996; Peciña and Berridge, 2005; Ambroggi et al., 2008). The NAcc activity would then promote social approach and attachment toward conspecifics that could subsequently elicit other behaviors, such as mating with the partner or huddling in the case of Cricetid rodents, tail twining in titi monkeys, and allogrooming in marmosets. Huddling, as mentioned earlier, is a behavioral signature heavily involved in Cricetid social bonding that is also present in NWP (Ruiz, 1990; Ågmo et al., 2012). In addition, mating is known to accelerate pair bonding both in Cricetid rodents (Williams et al., 1992; Insel et al., 1995a) and NWP (Evans and Poole, 1983; Mendoza and Mason, 1986; Mason and Mendoza, 1998), so brain regions likely involved in mating behavior, such as the MeA, BNST, mPOA, and the VMH (Li et al., 2017; Schnitzer et al., 2017; Turner et al., 2019) will interact with the NAcc or LS to mediate an association of mate cues and corresponding neural representation, with a rewarding valence. The LS is shown to be involved in social affiliation and pair-bond formation and maintenance in both rodents and titi monkeys. As mentioned before, the LS may act as a hub for social memory processing, reward and contextual information, and integrating relevant information for social behavior (Bielsky et al., 2005; Lukas et al., 2013; Wirtshafter and Wilson, 2020). While DA receptor expression patterns differ in the LS and NAcc between Cricetid rodents and NWP (Freeman and Young, 2016), evidence suggests that plastic changes in DA receptors in reward-related regions are necessary for pair-bond formation and maintenance. It is possible that for titi monkeys the LS is the network node that undergoes the DA-D_1_ receptor up-regulation needed for pair-bond maintenance, since the LS has both OT and DA receptor expression, and the role of the NAcc is not yet clear. On the other hand, common marmosets express OTR in the NAcc (Schorscher-Petcu et al., 2009). Several experiments suggest OT is a relevant modulator of social-reward in the marmoset (Smith et al., 2010; Cavanaugh et al., 2014), but the role of DA receptors in pair-bonding is unclear (Carp et al., 2019). Hence, while some structural similarities of nonapeptide expression with the titi monkey may imply shared neurobiological mechanisms in the marmoset, further research in marmosets is required to better understand the functional dynamics between brain regions underlying socio-sexual behavior. The VP also seems crucial for pair-bond formation and maintenance, probably by encoding the magnitude of the reward valence (Panagis et al., 1997; Smith et al., 2008; Ottenheimer et al., 2018) of the stimulus information provided from the LS and the NAcc. Monogamous rodents and monkeys also match in amygdala and cortical activity for both social affiliation and pair-bond formation, suggesting the integration of sensory stimulus information and social decision-making, respectively (Demas et al., 1997; Assadi et al., 2009; Rutishauser et al., 2015).

**TABLE 1:** Species-comparison of known SDMN brain region activity, circuit interactions, and neurotransmission on mating and monogamous behaviors.

	Mating	Social affiliation	Pair bond formation	Partner preference	Mate guarding	Pair bond maintenance
Prairie vole (*Microtus ochrogaster)*	**↑ c-Fos**^a^: mPFC, NAcc, AON, BLA, PVN, VP, BNST, mPOA **↑ eFC**^b^: mPFC-NAcc, mPFC-BNST, mPFC-SHy **↑ DA**^c^: NAcc **↑** **AVPr**^q^: VP	**↑ c-Fos**^b+,n^: VTA, LS, MeA, VP, BNST, AH, mPOA, ventrolateral VMH, PeV **↑ eFC**^b^: mPFC-NAcc ↑ **rsFC**^d^: ACC-NAcc-BLA-RSC-VTA-LS-MeA-VP, RSC-PVN, BLA-DG **↑** **AVPr**^r^: VP	**↑ rsFC**^d^: VP-LS-RSC, ACC-vHC **↑ D2R**^e^: NAcc **↑ OpR**^k^: NAcc **↑ AVPr**^q^: LS ↓ **rsFC**^d^: LS-dHC	**↑ rsFC**^d^: LS-VP **↑ DA**^c^: NAcc **↑ D2R**^e^: NAcc **↑ OpR**^k^: NAcc **↑ OTR**^o^: NAcc **↑** **AVPr**^r^: VP ↓ **rsFC**^d^: BLA-NAcc, LS-RSC ↓ **AVPr**^p^: RSC ↓ **ER**α^m^: MeA	**↑ c-Fos**^n^: LS, VP, VTA, BNST, mPOA, VMN, AH **↑ D1R**^e^: NAcc	**↑ rsFC**^d^: LS-mPFC-dHC, VP-VTA **↑ D1R**^e,c+:^ NAcc **↑ CB1**^a+^: NAcc ↓ **rsFC**^d^: BLA-NAcc-VTA, ACC-LS
Mandarin vole (*Microtus mandarinus*)	↓ **AR, T**^e+^:LS, MeA, mPOA, VMH	**↑ DA**^f+^: NAcc	↓ **D2Re**^f^*^+:^* NAcc	**↑ OT**^d+^ **↑ c-Fos**^d+^: BNST, LS, mPOA, PVN, SON, MD, VMH, MeA, CeA	*NA*	*NA*
Titi monkey (*Callicebinae* spp.)	*NA*	**↑ FDG**^h^: NAcc, LS, ACC, CeA, HC, PVN, SON **↑ OT**^s^*CPSTABLEENTER*↓ **OTR**^z^: HC ↓ **OpR**^v^	**↑ FDG**^f:^ NAcc, VP, mPOA, MeA, SON, LS **↑ D1R**^i:^ LS	**↑ AVP** ^x^	**↑ FDG**^j^: right LS, left PCC, left ACC **↓ FDG**^j^: MeA **↑ D1R**^u^	**↑ FDG**^g^: NAcc, VP **↑ D1R**^i^: LS
Common marmoset (*Callithrix* spp.)	*NA*	**↑ OT**^t^ **↓ D2R**^w^	*NA*	**↑ OT** ^t^	↓ **OT**^y^	*NA*

Behaviors shown are facilitated by the increase or decrease of activity of brain regions and specific types of neurotransmission.

**↑,** increase of; ↓, decrease of; NA, data not found by the time of this review.

AR, androgen receptor expression (male voles). AVP, arginine-vasopressin concentration level (exogenous and endogenous). AVPR, arginine-vasopressin receptor binding. c-Fos, c-fos protein expression (immunohistological detection correlates). D1R, dopamine D_1_ receptor binding. D2R, dopamine D_2_ receptor binding. DA, extracellular dopamine concentration (microdialysis detection). Efc, electrophysiological functional connectivity (low-frequency signal coherence correlates). ERα, estrogen receptor alpha binding. FDG, [^18^F]-fluorodeoxyglucose uptake (metabolic activity correlates). OpR, opioid receptor binding. OT, oxytocin concentration level (exogenous and endogenous). OTR, oxytocin receptor binding. rsFC, resting-state functional connectivity (low-frequency fMRI BOLD-signal correlates). T, testosterone expression (male voles, immunohistological).

ACC, anterior cingulate cortex. BLA, basolateral amygdala. BNST, bed nucleus of the stria terminalis. CeA, central amygdala. DG, dentate gyrus. HC, hippocampus. dHC, dorsal hippocampus. MeA, medial amygdala. MOB, main olfactory bulb. LS, lateral septum. mPFC, medial prefrontal cortex. mPOA, medial preoptic area. NAcc, nucleus accumbens. PCC, posterior cingulate cortex. PeV, periventricular nucleus of the thalamus. PVN, paraventricular nucleus of the hypothalamus. RSC, retrosplenial cortex; SON, supraoptic nucleus. SHy, septohypothalamic nucleus. vHC, ventral hippocampus. VMN, ventromedial nucleus of the hypothalamus. VP, ventral pallidum. VTA, ventral tegmental area. ^a^(Lim and Young, 2004), ^b^(Amadei et al., 2017), ^c^(Aragona et al., 2003; Gingrich et al., 2005), ^d^(López-Gutiérrez et al., 2021), ^e^(Aragona et al., 2006), ^f^(Bales et al., 2007), ^g^(Maninger et al., 2017b), ^h^(Arias Del Razo et al., 2020), ^i^(Hostetler et al., 2017), by D1 receptor antagonist [11C]-SCH2339 binding detection, ^j^(Maninger et al., 2017b), ^k^(Burkett et al., 2011; Resendez et al., 2013, 2016); ^l^(Lei et al., 2010), ^m^(Lambert et al., 2021), ^n^(Gossman et al., 2021), ^o^(King et al., 2016), ^p^(Ophir et al., 2008b), ^q^(Liu et al., 2001; Lim and Young, 2004), ^r^(Pitkow et al., 2001; Lim and Young, 2004), ^s^(Witczak et al., 2018), ^t^(Cavanaugh et al., 2014, 2018a), ^u^(Rothwell et al., 2019), ^v^(Ragen et al., 2015b), ^w^(Carp et al., 2019), ^x^(Jarcho et al., 2011), ^y^(Cavanaugh et al., 2018b), ^z^(Baxter et al., 2020)), ^a+^(Borie et al., 2022), ^b+^(Cushing et al., 2003), ^c+^(Lei et al., 2017), ^d+^(Jia et al., 2008), ^e+^(He et al., 2013), ^f+^(An et al., 2013).

**FIGURE 1:**
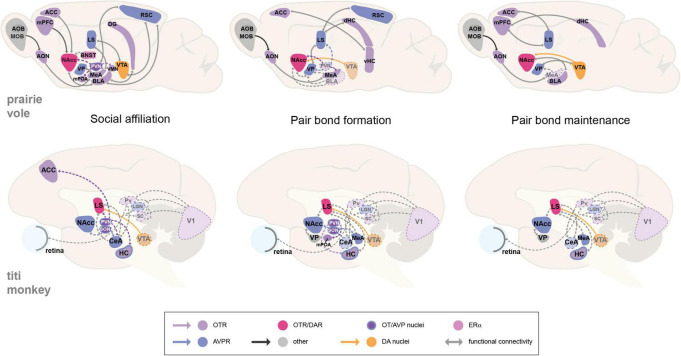
Proposed neural circuits of monogamous behavior in the prairie vole and the titi monkey. Illustrations show sagittal views of the prairie vole and titi monkey brain that propose neural circuit models for social affiliation, pair bond formation, and pair bond maintenance. The behaviors imply the recruitment of reward-modulating areas with social-salience processing regions, producing an association between a social cue or stimuli with a reward encoding and response. The activation of particular brain areas may be context-dependent, hence the dashed connecting lines and dashed brain region outlines that show hypothesized interactions that are likely involved, but have yet to be demonstrated for these circuits. OT, oxytocin; AVP, arginine-vasopressin; DA, dopamine; ERα, estrogen receptor alpha; OTR, oxytocin receptor; AVPR, arginine vasopressin receptor; ACC, anterior cingulate cortex; AOB, accessory olfactory bulb; AON, anterior olfactory nucleus; BLA, basolateral amygdala; BNST, bed nucleus of the stria terminalis. CeA, central amygdala. DG, dentate gyrus. HC, hippocampus. dHC, dorsal hippocampus. MeA, medial amygdala. MOB, main olfactory bulb. LGN, lateral geniculate nucleus. LS, lateral septum. mPFC, medial prefrontal cortex. mPOA, medial preoptic area. NAcc, nucleus accumbens. Pv, pulvinar. PVN, paraventricular nucleus of the hypothalamus. RSC, retrosplenial cortex. SC, superior colliculus. SON, supraoptic nucleus. VP, ventral pallidum. vHC, ventral hippocampus. VTA, ventral tegmental area. V1, primary visual cortex.

The involvement of nonapeptide (OT or AVP) neurotransmission is also shown for social affiliation in both mammalian groups by increased activity in the PVN and SON, which is expected due to their involvement in modulating social behavior (Johnson et al., 2017; Arias Del Razo et al., 2020). Nevertheless, the study of neural circuits has shown that while some regions may seem to be more functionally relevant by acting as hubs or centers of co-localization for specific receptors, all associated brain regions may substantially contribute to encoding a neural representation of the partner that is associated with the pair bond, and should accordingly activate for an appropriate, context-dependent behavioral decision and response. The observed increase and decrease in the activity of specific brain regions and associated receptors, in order to facilitate a particular social or sexual behavior, emphasizes the complexity of the modulation of socio-sexual circuits and the need to expand research on the involvement of multiple brain areas and their relationship with the expression of particular behaviors (Figure 1). It is also important to consider how reproductive cycles and the life span of each species impact pair bond formation and maintenance. For instance, bond formation in monogamous rodents can take as little as 6 h (Williams et al., 1992), and once paired, they mate and give birth to new litters constantly. On the other hand, pair bond formation in NWP may take days to weeks, and unlike the rate of affiliative behavior, rates of sexual activity in NWP tend to decline after pairing (Schaffner et al., 1995; Ågmo et al., 2012). Thus, distinguishing context-specific circuit activity between social and sexual behaviors, and how they influence social monogamy in each taxon, should be addressed in future studies. Next, we will discuss relevant factors known to modulate the neural circuits of monogamous behavior and their implications for mating and social bonding.

### Factors modulating the neural circuitry of monogamous sexual behavior

#### Genetic and epigenetic variability

In the sections above, we discussed how genetic and transcriptomic factors are generally involved in monogamous behavior. However, differences in the gene, protein expression, and epigenetics have been recognized to influence socio-sexual behavior at species, individual, and sex levels.

A considerable number of studies have shown evidence of these phenomena. In prairie voles, a functional microsatellite polymorphism of the AVPr1a gene that was discovered to be species-specific (Hammock and Young, 2004) was shown to vary individually and to correlate with social behavior in males (Hammock et al., 2005), though the impact of this particular polymorphism over certain behaviors has been debated (Ophir et al., 2008a; Solomon et al., 2009). However, mRNA interference of the expression of this receptor is known to impair partner preference (Barrett et al., 2013), while such expression has been enhanced by inducing an overexpression of AVPr in the VP of a non-monogamous vole species (Lim et al., 2004). Higher levels of AVPr expression have also been related to higher sexual fidelity in male prairie voles (Ophir et al., 2008b) and aggression in California mice (Bester-Meredith et al., 1999).

In the case of OT, a recent study has suggested that OTR expression variability may be attributable to polymorphisms in the OTR gene (Inoue et al., 2022). Higher sexual fidelity in male voles has also been correlated with higher receptor density in the NAcc (Ophir et al., 2012; King et al., 2016), OTR density in the insula predicted mating success, and OTR density in the hippocampal region predicted patterns of socio-spatial memory (Ophir et al., 2012). In NWP, one report described that higher OTR binding in the hippocampal formation, specifically the presubiculum, is correlated to parenthood status and partner affiliative behavior in the titi monkey (Baxter et al., 2020). Parental and alloparental care are also reportedly influenced by nonapeptide receptor density in the prairie vole (Olazábal and Young, 2006), California mice (Perea-Rodriguez et al., 2015), and mandarin voles (*Microtus mandarinus*) (Yuan et al., 2019).

Interestingly, OT may have a dose-dependent effect on social behavior, and treatments that increase central OT may have unexpected results. Chronic intracerebroventricular OT treatment in mandarin voles reduced sociability and reduced OTR levels in the NAcc and MeA (Du et al., 2017), also inducing alterations in AVPr and DA receptors in the same region. Evidence shown by the latter mandarin vole studies suggests that nonapeptide modulation, as proposed earlier, may be subtle and may act in balance with other neurotransmitter systems. Accordingly, differences in levels of estrogen-receptor α (ERα) expression in the MeA, mPOA, and the VMN are also associated with monogamous behavior and parental care in mandarin voles (Wu et al., 2011).

With respect to OT variants and their impact on behavior, interesting discoveries have been made. Phylogenetic evidence has shown not only OT genetic sequence differences across closely related species, but also a functional cross-talk between AVP and OT, and structural variations in the expressed nonapeptides that could subsequently impact their involvement in neural circuits (French et al., 2016; Pierce et al., 2020). This is the case for the marmoset variant of the OT ligand (Pro^8^-OT), which has a proline substitution at the eighth amino-acid position instead of the consensus mammalian variant of the OT ligand (Leu^8^-OT). Thus, when marmosets are treated with OT Pro8 variant, robust behavioral effects like facilitation of fidelity with a long-term partner and partner social preference are observed (Cavanaugh et al., 2014, 2015; Mustoe et al., 2015). Although the consensus mammalian variant does have a reported effect on socio-sexual behaviors in marmosets (Smith et al., 2010; Cavanaugh et al., 2018a), differences in OT variants have to be taken into account experimentally. Also, functional limitations over OT sequence differences have to be considered when comparing socio-sexual behavior between taxa.

In addition to levels of neuroreceptor expression, transcriptomic changes have been detected as a result of monogamous socio-sexual behavior. It has been found that the NAcc undergoes significant transcriptomic changes due to pair bond formation and maintenance in the prairie vole, including sex-specific changes in protein turnover, DNA transcription, chromatin modifications, mitochondrial dynamics, and neurotransmission (Duclot et al., 2022). Another study reported that genes involved in transcription regulation, neuron structure, and synaptic plasticity were differentially expressed between prairie and the promiscuous meadow voles after mating (Tripp et al., 2021). While these studies have shown that cohabitation and pair bonding depend on transcriptomic mechanisms and modifications of the neural structure, it would be interesting to analyze and compare molecular changes underlying the expression of other socio-sexual behaviors in mammalian species.

Further, epigenetic mechanisms understood as heritable changes in gene expression that are not due to alterations in DNA sequence have been reported to be relevant in modulating social and sexual behavior. Epigenetic mechanisms may include histone modifications, chromatin remodeling, DNA methylation, and microRNA (Zhang et al., 2020). Wang and collaborators (Wang et al., 2013) evaluated the epigenetic mechanisms mediating pair bonding in prairie voles. The intracerebroventricular administration of histone deacetylase inhibitors TrichoStatin A or sodium butyrate were found to increase histone acetylation in the NAcc of female voles, which in turn enhanced partner preference even in the absence of mating. Similar epigenetic modifications were observed in females that developed partner preference after mating, such as higher H3K14 acetylation levels in the OTR and AVPr1a promoters in the NAcc. Likewise, TrichoStatin A treatment promoted partner preference in male voles in the absence of mating, increasing OTR gene expression in the NAcc in comparison to sexually naïve males (Duclot et al., 2016).

Another interesting study on prairie voles reported that low levels of early care, with care being defined as constant physical contact, were shown to correlate with *de novo* DNA methylation at specific regulatory sites on the OTR gene in the NAcc (Perkeybile et al., 2018). An increased level of methylation was negatively correlated with OTR expression and binding in the NAcc, which in turn has been previously associated with reduced levels of maternal care. Thus, epigenetic modifications have been shown as relevant factors in shaping the neural circuits of socio-sexual behavior and in generating behavioral variability in the prairie vole. Expanding research over other taxa would likely reveal crucial findings on whether epigenetic regulation is a widespread mechanism in modulating the expression of mammal monogamous socio-sexual behavior.

The aforementioned studies suggest that behaviors associated with social monogamy show variability depending on the genotype of the individual, inducing a differential circuit-level modulation that in turn generates behavioral diversity and different mating tactics that may enable a fluid transition in population changes. Also, monogamous behaviors such as pair-bond formation induce profound neurobiological changes at several levels, closing the gap in understanding the mechanisms underlying this behavior. Moreover, deepening the study of translational genetic and epigenetic factors over behaviors associated with social bonding and social monogamy could have therapeutic potential by harnessing the fundamental mechanisms underlying the modulation of social behavior.

#### Early life experience: Parental nurturing and parental deprivation

Early social experiences (e.g., interactions with parents or peers) at critical developmental stages can profoundly shape the social behavior of an individual later in life (Tzanoulinou and Sandi, 2017). The impact of paternal behavior has been explored in socially monogamous rodents that engage in the biparental care of their offspring. The presence of both mother and father as early caregivers allows studying the impacts of the variation of paternal and maternal care on various developmental aspects in offspring, including behaviors unique to socially monogamous mammals, such as pair bonding.

In prairie voles, males and females contribute equally to offspring care (Thomas and Birney, 1979), and mothers do not modify the amount of licking and grooming toward pups if fathers are removed; hence, pups receive less licking and grooming when raised by their mother alone (Ahern and Young, 2009; Valera-Marín et al., 2021). The monoparental upbringing of prairie voles has been shown to have no influence on the display of sexual behavior of adult males (Valera-Marín et al., 2021), but it does delay partner preference formation in adult males and females (Ahern and Young, 2009). Thus, the behavioral differences between voles of different family structures seem to center on the processing of social reward.

This was shown when it was reported that cohabitation with mating induced a reward state in biparentally but not monoparentally raised voles evaluated in a conditioned place preference test (Valera-Marín et al., 2021). Furthermore, males raised by both parents, but not by a single mother, show an increase in DA turnover (3,4-dihydroxyphenylacetic acid/DA and homovanillic acid/DA) in the NAcc. Together, these data suggest that sexual interaction in monoparental males is not as rewarding in comparison to biparental-raised male voles, which may modify their mating tactics toward a less monogamous strategy (Valera-Marín et al., 2021). Moreover, the substitution of fathers for alloparents demonstrated that paternal absence affects pair-bond formation in female offspring *via* reduced quantity of care but affects pair-bond formation in male offspring by means of a missing paternal quality (Rogers and Bales, 2020). Parental care has also been shown to alter nonapeptide neurochemistry in offspring. OTR gene polymorphisms and parental rearing have been shown to interact, resulting in distinct social-behavior phenotypes (Ahern et al., 2021). Compared to biparentally reared individuals, males raised by a single mother show lower densities of OTRs in the CeA, CP, and the NAcc, whereas females raised in the absence of a father show greater densities of AVPr in the MeA and VMN (Rogers et al., 2021). This study shows that paternal deprivation induces sex-dependent changes in the expression of OT and AVPr in several social processing brain nuclei. Alterations in the levels of expression of neurotrophic factors and their epigenetic regulation in the HC have also been reported as a result of paternal deprivation (Tabbaa et al., 2017). Social factors beyond the neonatal environment (e.g., during juvenile and adolescent development) can also shape adult social behavior. For example, male voles raised by a single mother and weaned into social isolation are more likely to establish stronger partner preferences, and when treated intranasally with OT during juvenile development, they exhibit substantial huddling behavior (Prounis and Ophir, 2019). The findings mentioned above suggest that parental care variation in the early social environment of monogamous rodents can induce changes in attachment behaviors by influencing sex-dependent changes in OT, AVP, and DA activity in key brain regions involved in pair-bond formation and the expression of other monogamous social behaviors.

The effects of parental care and early social deprivation on the socio-sexual behavior of the mandarin vole, a cricetid rodent known to also display biparental care, have been evaluated in several studies. Paternal deprivation has shown to inhibit the development of social recognition (Cao et al., 2014), reduce sociability and parental behavior in adult offspring (Jia et al., 2011; Yu et al., 2015), delay pair-bond formation (An et al., 2013), and increase anxiety-like behavior (He et al., 2018, 2019) and aggression (Yu et al., 2012). At a molecular level, decreased levels of OTR in the NAcc (An et al., 2013; Cao et al., 2014) and reduced hippocampal glucocorticoid receptor levels have been found (Wu et al., 2014) whereas NAcc serum corticosterone levels have been reported to increase (Yu et al., 2015). Thus, in this vole species, nonapeptide and DA interaction are necessary for the modulation of social behavior. When pre-weaned pups are treated with OTR antagonists, central DA is decreased and DA receptor expression is reduced in the NAcc, altering pup attachment behavior (He et al., 2017). Furthermore, OT administration rescued the paternal deprivation-induced changes in anxiety-like behaviors and social preferences (He et al., 2019). In the latter study, it was also shown that the release of OT in the PVN-prelimbic pathway through optogenetic stimulation was able to reduce anxiety-like behavior and restore deficits in social behavior. And as explained elsewhere, ERα is also involved in regulating socio-sexual behavior in mandarin voles. Paternal deprivation has been reported to reduce estradiol serum (He et al., 2018) and ERα expression in the BNST, MeA, mPOA, VMN, and the NAcc (Jia et al., 2011; Cao et al., 2014; Feng et al., 2019). The studies mentioned suggest that OT, DA, and estradiol modulation are influenced by parental care, with the NAcc representing the main region of interaction that potentially regulates attachment and parental behavior in offspring of mandarin voles.

In the California mouse (Peromyscus californicus), it is also known that the presence of the father in the nest is critical for offspring survival in the wild (Gubernick and Teferi, 2000). Therefore, variations in parental care can alter offspring behavior. Accordingly, the amount of licking and grooming received by pups, which is diminished if the father is absent, may impact cognitive performance, particularly spatial learning, when pups reach adulthood (Bredy et al., 2004). Also, decreased levels of early paternal care have been associated with increases in AVP immunoreactivity in the PVN of the hypothalamus and increased basal corticosterone levels (Frazier et al., 2006). Other studies that have addressed the impact of paternal care in the California mouse have mainly focused on aggression (Bester-Meredith and Marler, 2003; Frazier et al., 2006; Becker et al., 2010) and stress (Kowalczyk et al., 2018). However, it is very likely that behavioral neural circuits in the California mice are influenced by parental care, though more studies specifically addressing the influence over socio-sexual behavior are necessary.

In the case of NWP, current literature allows the proposal of some hypotheses on how neural circuit modulation is influenced by parental care. For instance, infant titi monkeys show distress-like behavior when separated from their father, which is their main attachment figure (Mendoza and Mason, 1986; Ragen et al., 2012; Spence-Aizenberg et al., 2016). However, when subjected to an adverse early experience, they were less likely to maintain proximity to their father and exhibited more exploratory behavior during the separation condition (Larke et al., 2017). Also, the type of attachment an infant titi monkey displays with its father may be indicative of the type of attachment it will share with its adult pair mate (Savidge and Bales, 2020). This is consistent with what has also been observed in several vole species, and it is likely that the reward components of the socio-sexual circuit in NWP, such as the LS, will undergo functional and modulation changes with other regions of the SDMN as a consequence of parental care variation, modifying reward valences and behavioral responses related to social affiliation and interaction.

A recent study in titi monkeys suggests that OT treatment in juveniles can induce long-term changes in the sensitivity to socially relevant cues, with female titi monkeys having a greater response to treatment due to greater glucose uptake in the whole brain and in socio-sexually relevant regions (Razo et al., 2022). In marmoset monkeys the effects of paternal deprivation that may alter socio-sexual functional networks have also been documented. Daily parental deprivation during infancy has been reported to induce endocrine stress responses, anhedonia, and long-term alterations in hippocampal function (Dettling et al., 2002, 2007; Pryce et al., 2004; Law et al., 2009a,b). Studies specifically focused on the impact that parental care has on the neural circuit of the pair bond in adult offspring in NWP would certainly shed more light onto this matter.

Indeed, parental influence over the neurobiological development of offspring is a relevant factor that will impact mammal socio-sexual behavior on a long-term basis (Champagne and Meaney, 2007). It is enticing to extend to other taxa a proposition first made on prairie vole mating behavior by our research group: parent availability and level of care may shape offspring behavior to enable a better adaptation for future socializing and mating with other conspecifics. A fluctuating population will not only impact parent availability and paternal care over a particular generation but will also affect mate availability. Hence, neural circuits of social behavior may be tuned from an early age to adapt to the socio-sexual strategy that will be a better fit for survival (Valera-Marín et al., 2021).

#### Social isolation and partner separation

Considering the importance of social behavior in monogamous mammals for both reproduction and parental nurturing, social isolation can act as an acute stressor that induces significant alterations in overall health (Hawkley et al., 2012) and alters several brain systems involved in social behavior (Ross et al., 2019; Donovan et al., 2020). For example, prairie voles that are socially isolated after weaning show increased anxiety-like behavior (Pan et al., 2009), and brain tissue analyses reported increased mRNA expression of OT, AVP, corticotropin-releasing factor (CRF) and TH in the PVN. In addition, social isolation at an early stage of development can also induce impairments in social behavior, similar to what is observed with paternal deprivation. When voles are socially deprived before weaning and are socially isolated after weaning, they show impairments in social recognition and discrimination when they reach adulthood, with altered OTR expression in the LS (Prounis et al., 2015). Moreover, voles deprived before weaning that were later socially enriched after weaning had no signs of altered social recognition, suggesting that social enrichment at a later age can rescue pre-weaning social deprivation. Still, these voles were shown to have behavioral and neurobiological differences from voles that did not have any social deprivation. Therefore, conditions such as social isolation can alter neural circuits related to social behavior that will potentially impact future socio-sexual interactions, including pair bond formation.

Partner separation and partner loss in monogamous mammals are also known to induce similar signs of distress and adverse physiological effects, potentially altering the neural circuits underlying monogamous social bonding. In the prairie vole, disruption of the pair bond induces depressive-like and anxiety-like related behaviors, cardiac dysregulation and autonomic dysfunctions (Bosch et al., 2009; McNeal et al., 2014; Sun et al., 2014; Osako et al., 2018; Feng et al., 2021).

Male mandarin voles are also reported to display anxiety-like and depressive-like behaviors when separated from their partner (Feng et al., 2021). In addition to acute behavioral responses, changes at a molecular level have also been documented. There is evidence that bond disruption (4 days of separation) in the prairie vole activates CRF receptors, which in turn may reduce OT mRNA in the PVN and decrease NAcc OTR, impairing OT signaling in the NAcc (Bosch et al., 2009, 2016). Prairie vole mothers also show increased CRF mRNA receptors when they are abandoned by their male partners (Bosch et al., 2018). Interestingly, it appears that temporal thresholds are important in modulating behavioral responses toward partner loss. Male prairie voles will show preference for their partner after two, but not 4 weeks of separation (Sun et al., 2014). Nevertheless, losing their partner still induced anxiety and depressive-like behaviors, and altered density of OT, AVP, and corticotrophin-releasing hormone positive cells in the PVN were detected. Thus, this study suggests that prairie voles may have the capability of forming new bonds but may not leave unscathed from the bond breakage, and the respective process will involve stress-like responses and neurobiological changes.

In NWP, behavioral, physiological and circuit-level changes have also been documented after partner separation. Short-term partner separation of pair-bonded titi monkeys induced FDG uptake changes in the LS, PVN, PAG, and the cerebellum, also increasing OT levels in cerebrospinal fluid (Hinde et al., 2016). Long- term separation also induced FDG uptake changes in the CeA and increased cerebrospinal fluid OT levels. Overall, the changes described in the study were attributed to nonapeptide and opioid system reactions toward partner separation. In marmoset monkeys, treatment with an OT antagonist produced less time spent in proximity with their pair mate upon reunion following a long-term separation challenge, suggesting that OT may act as a bond modulator (Cavanaugh et al., 2018a). Similarly, it has also been proposed that OT release during social deprivation in prairie voles has a buffering function in response to the induced stress (Grippo et al., 2007).

Overall, these studies suggest that short-term separation may cause acute, distress-like responses in monogamous mammals, but when separation occurs for a longer period, signifying permanent unavailability of the partner, breakage of the bond may potentially happen. The breakage of the bond suggests alteration of the socio-sexual circuits, in which the neuroreceptor systems involved may submit to a reconfiguration of the rewarding valence associated with the neural representation of the partner. This reconfiguration process would likely produce discomfort, given the participation of the circuits involved in reward-seeking behavior and the behavior observed. However, this process may result in changes that enable the possibility of forming a new pair bond with another sexual partner, which in some circumstances, such as premature death or an intentional abandonment, may result in a better mating strategy. Further characterization of the functional changes associated with partner loss and bond disruption in neural circuits of monogamous mammals would aid in understanding the neurobiological processes that also occur in humans when social bonds are disrupted or lost.

## Conclusion

Overall, recent studies suggest that the neural circuits of social monogamy are more complex than previously thought, involving a dynamic interplay of brain structures and neurotransmitter systems. Monogamous behavior, which is most likely a convergent evolutionary phenomenon, is similar across mammal species and shares neurobiological mechanisms. In these mechanisms, the activity of brain regions in the neural circuits of socio-sexual behavior functionally overlaps. Moreover, dopamine, OT and AVP seem crucial for the expression of behaviors related to social monogamy. Nonapeptide modulation, however, is not evident for all species and further research is necessary to extend this hypothesis and assessing their role with other neurotransmitter systems and other molecular mechanisms will be relevant for the field. Increasing evidence shows that AVP and OT activity may not be the sole modulators of monogamous behavior, which may partly explain the neurobiological diversity observed at the individual level and in different taxa. Reports studying circuit-level dynamics also suggest that neuroreceptor systems and brain regions interact in a temporal, context-dependent manner that may relate to the expression of a particular behavior. Also, neural circuits related to social monogamy are being modulated by an increasing number of factors, which should be considered in order to deepen our understanding about socio-sexual behavior. These factors not only influence mate choice and reproductive success of the individual but may impact at the population level and intertwine with the ecological and environmental context. The ability that new techniques have shown in associating specific functional interactions of the brain with behavior is a powerful tool that enables a more detailed characterization of the socio-sexual circuits, and potentially in the development of more targeted therapeutics related to social and sexual health.

## Author contributions

ML-G, SM-C, and WP: conceptualization, writing original draft, and editing. SA: conceptualization and editing. All authors contributed to the article and approved the submitted version.
